# Human Decidual Mesenchymal Stem Cells Obtained From Early Pregnancy Improve Cardiac Revascularization Postinfarction by Activating Ornithine Metabolism

**DOI:** 10.3389/fcvm.2022.837780

**Published:** 2022-02-11

**Authors:** Kegong Chen, Long Bai, Jingtong Lu, Wei Chen, Chang Liu, Erliang Guo, Xionghai Qin, Xuan Jiao, Mingli Huang, Hai Tian

**Affiliations:** ^1^Department of Cardiovascular Surgery, The Second Affiliated Hospital of Harbin Medical University, Harbin, China; ^2^Key Laboratory of Myocardial Ischemia, Ministry of Education, Harbin Medical University, Harbin, China; ^3^Future Medical Laboratory, The Second Affiliated Hospital of Harbin Medical University, Harbin, China; ^4^Department of Chest Surgery, Affiliated Cancer Hospital and Institute of Guangzhou Medical University, Guangzhou, China; ^5^Department of Chest Surgery, The Third Hospital of Xiamen, Xiamen, China; ^6^Department of Gynecology and Obstetrics, The First Affiliated Hospital of Harbin Medical University, Harbin, China

**Keywords:** ischemic heart disease, decidual mesenchymal stem cells, bone marrow mesenchymal stem cells, ornithine decarboxylase, revascularization, heart remodeling

## Abstract

**Background:**

Compared with bone marrow mesenchymal stem cells (BMSCs), decidual mesenchymal stem cells (DMSCs) are easy to obtain and exhibit excellent angiogenic effects, but their role in cell transplantation after myocardial infarction (MI) remains unclear.

**Methods:**

BMSCs and DMSCs were harvested from healthy donors. The effects of both cell types on angiogenesis were observed *in vitro*. Metabonomics analysis was performed to compare different metabolites and screen critical metabolic pathways. A murine model of acute myocardial infarction (AMI) was established, which was randomized into five groups (control, BMSC, DMSC, DMSC + ODCshRNA and BMSC + ODC consisting of 50 animals, equally divided into each group). The therapeutic effect of DMSCs on MI in rats was assessed based on neovascularization and cardiac remodeling.

**Results:**

DMSCs exhibited a better angiogenic effect on human umbilical vein endothelial cells (HUVECs) than BMSCs *in vitro*. In addition, ornithine metabolism, which is associated with vascularization, was significantly increased in DMSCs. The transplantation of DMSCs in the rat MI model significantly enhanced angiogenesis of the infarct border area and improved cardiac remodeling and dysfunction postinfarction compared with BMSCs. Furthermore, inhibition of ornithine metabolism by silencing ornithine decarboxylase (ODC) in DMSCs partly abolished the benefits of DMSC transplantation.

**Conclusion:**

Compared with BMSCs, DMSCs exhibited better efficacy in improving revascularization and heart remodeling post-MI via the activation of ODC-associated ornithine metabolism.

**Graphical Abstract:**
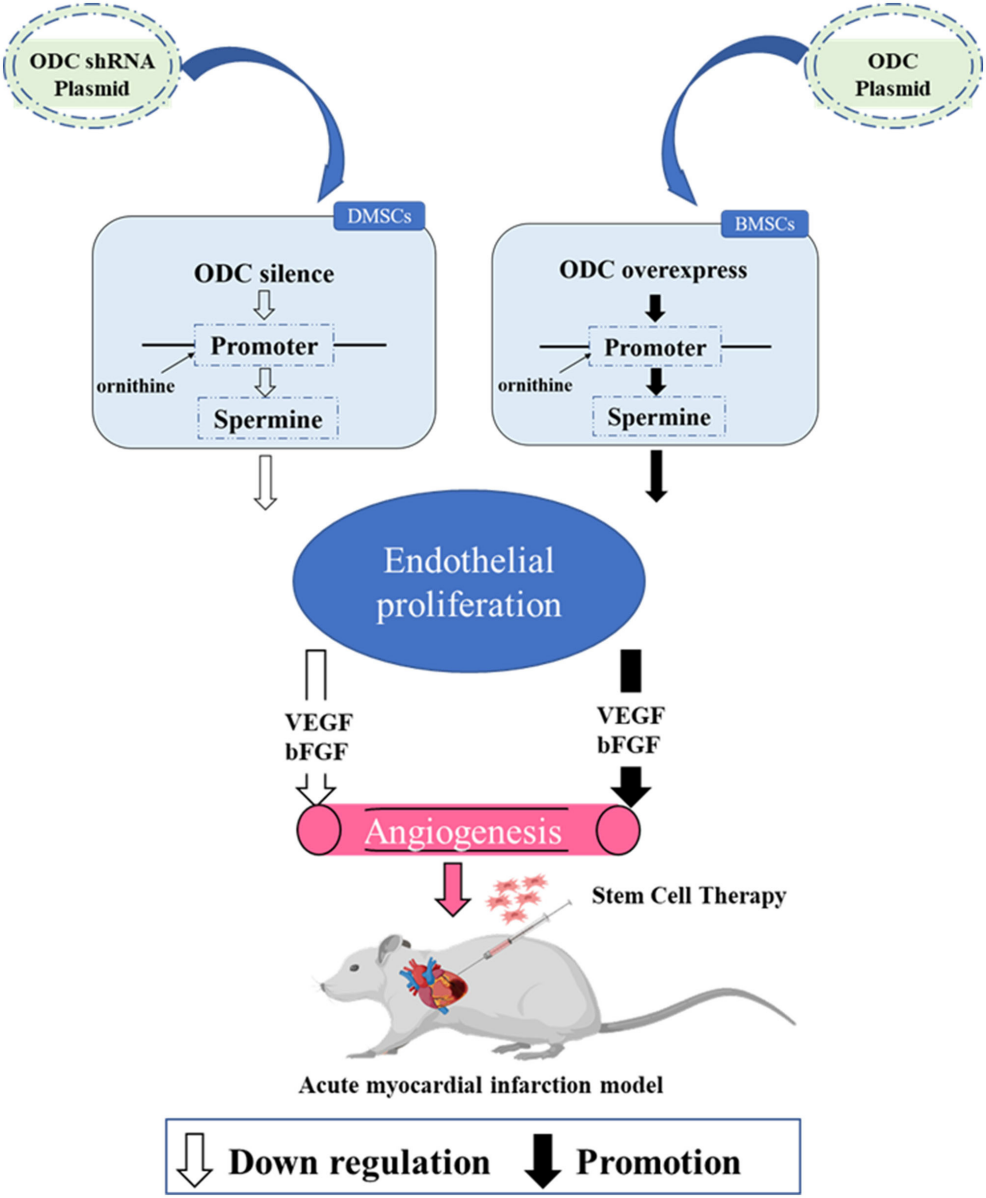
Silencing ODC in DMSCs inhibited spermine secretion, weakened endothelial cell proliferation and the expression of VEGF and bFGF, resulting in decreased angiogenesis. Overexpression of ODC in BMSCs promoted spermine secretion, enhanced endothelial cell proliferation and the expression of VEGF and bFGF, resulting in increased angiogenesis. Transplantation of DMSCs can better improve angiogenesis after myocardial infarction by activating ornithine metabolism.

## Introduction

Despite the advances in reperfusion and drug therapy, acute myocardial infarction (MI) remains one of the major causes of cardiovascular death (1). It is well known that bone marrow mesenchymal stem cells (BMSCs) could be utilized as a feasible candidate for cell transplantation after MI in view of their plasticity *in vivo* (2). However, BMSCs application did not show a satisfactory therapeutic effect on tissue repairment in clinical trials partly due to insufficient supply, limited proliferation capacity and aging (3). Decidual mesenchymal stem cells (DMSCs) from early pregnancy tissue have been found to proliferate significantly to promote vascular regeneration in severe preeclampsia, suggesting it may be a favorable potential target to treat ischemic heart disease (4). Decidual tissue harbors a large number of mesenchymal stem cells (MSCs), which are easy to obtain, exist in decidual tissue. In addition, the organizational source is so extensive that it is very likely to be an excellent seed cell in the renewable medical field for the treatment of ischemic heart disease.

Wang et al. reported that compared with BMSCs and adipose-derived mesenchymal stem cells (ADMSCs), human endometrium-derived mesenchymal stem cells (EnMSCs) were conferred a superior ability of cardioprotection and supports of enhanced microvessel density. The secreted exosomes expresses high level of miR-21, suggesting that miR-21 enhances cell survival through PTEN/Akt signaling pathway (5). Metabolomics is an emerging approach to comprehensively reveal the metabolic status of organisms correlating knowledge regarding genotype with phenotype. Metabolomics has been demonstrated to be an important tool for the discovery of biomarkers and potential drug treatment targets in cardiovascular disease (6). In addition to reflecting the disease phenotype, metabolites are implicated in the pathophysiological processes of numerous diseases. Thus, metabolomics may be beneficial for understanding both normal physiology and pathophysiology (7). Screening the differences in metabolite profiles between DMSCs and BMSCs might provide novel insights into the underlying mechanisms of DMSC transplantation (8). Previous studies have reported that active ornithine metabolism is related to tumor invasion and angiogenesis (9).

In this study, we found that ornithine and spermine, which were regulated by ODC, were the main mechanisms the angiogenesis of HUVECs. We also confirmed that DMSCs could better improve angiogenesis and cardiac remodeling post-MI *in vivo* via ODC-associated ornithine metabolism compared with BMSCs.

## Materials and Methods

### Isolation and Culture of Human DMSCs and BMSCs

Human decidual samples were collected from 20- to 30-year-old healthy women undergoing early pregnancy abortion in the clinic. Each sample was rinsed with saline, and then stored in a sterile bottle of a fully medium with 0.1% Penicillin and streptomycin until it was processed in the laboratory (<2 h later). All samples were rinsed for three times with sterile PBS, and then cut into 1–2 mm3 pieces, which were treated by 0.2% IV-type collaborate (Yeasen, Shanghai, China) and 0.25% trypsin. Ethylenediamine tetracetate (Trypsin EDTA; Gibco, Gaithersburg, MD, USA) covered the tissue, water bath (37°C/150 rpm) shaked it for 1 h subsequently. Cell digestion was filtered by a 200 μm nylon mesh (BD Falcon, San Jose, Ca, USA), which was cultured in DMEM/F12 (Hyclone, Southlogan, Utah, USA) medium containing 10% FBS (FBS; Sciencell, Sandiego, CA, USA) to terminate the enzymatic reaction. After being separated by centrifugation (1,000 rpm/5 min), cells were resuspended in 4 ml DMEM/F12 medium containing 10% FBS, and cultured in an incubator of 37°C, 5% CO2. On the second day, DMSCs were observed in the culture bottle (10). Similarly, BMSCs were also obtained from female patients (20–30 years old) who needed median thoracotomy in cardiac surgery, stored into a bottle pre-filled with heparin later, quickly transferred to the laboratory for treatment. An equal volume of lymphocyte separation solution was mixed with bone marrow by centrifugation at 2,500 × rpm for 20 min. BMSCs, the mononuclear cells layer, were obtained and washed twice. DMSCs and BMSCs were seeded in DMEM/F12 (HyClone) supplemented with 10% FBS (ScienCell) and cultured at 37°C in an atmosphere containing 5% CO_2_ (10). Third-generation DMSCs/BMSCs were used for the following experiments.

All participants provided informed consent, and all procedures in the study were conducted in accordance with the ethical standards of the First/Second Affiliated Hospital of Harbin Medical University and the principles of the Helsinki Declaration.

### Cell Morphological Observation and Phenotypic Identification

The morphology of both MSCs was observed under a light microscope (Leica, Germany). As for cell identification, 2 × 10^6^ BMSC and DMSC cell suspensions were fully mixed with CD73-PerCP, CD90-FITC, CD105-APC, CD34-FITC and CD45-PerCP antibodies for 30 min followed by detection via flow cytometry (BD, USA).

### Comparison of the Proliferation and Cloning Ability of DMSCs and BMSCs

DMSCs and BMSCs at an initial density of 1 × 10^3^ were cultured for 12, 24, 48, and 72 h in 96-well plates. Cell Counting Kit-8 (CCK-8; Dojindo, China) was utilized to detect proliferation ability according to the manufacturer's instructions (11).

DMSCs and BMSCs with initial densities of 300, 600, and 900/well were cultivated in 6-well plates and cultured for 2 weeks. After being fixed with 4% paraformaldehyde, the cells were covered with crystal violet staining solution for 10 min. A colony containing ≥20 cells was considered as a clonal colony.

### Metabonomic Analysis of DMSCs and BMSCs

The supernatants of DMSCs and BMSCs were deproteinized with 100 μL acetonitrile in centrifuge tubes, vortexed for 2–3 min, placed on ice water for 20 min, and then centrifuged at 12,000 rpm, 4°C for 15 min, which were transferred into another tube and centrifuged again with the mentioned conditions. At last, 200 μL supernatants were transferred into the sample bottle for LC-MS analysis. A 10 μL aliquot of the sample was injected into the ZORBAXSB-C18 column (AgilentTechnologies, INC) (12). Mass spectrometry analysis was assessed by using an Agilent Agilent 6,530-QTOF/MS instrument (Agilent Technologies) in both ESI + and ESI– modes. Finally, metabolic pathway enrichment was performed by using MetaboAnalyst.

### Plasmid Construction and Transfection

Plasmid containing ODC shRNA or ODC sequences was transfected to inhibit or up-regulate ODC in DMSCs and BMSCs, respectively. Cells transfected with empty plasmids were regarded as the control group. Plasmid transfection was conducted via Lipofectamine (Invitrogen, USA) following the manufacturer's instructions (13). The ratio of transfection reagents to plasmid was 2 μl:1 μg. Western blot analysis was used to assess ODC protein levels as is previously reported (14). After 72 h culture, Transfected cells with GFP expression were visualized with fluorescence microscope (Leica, Germany).which was used to identify transfected cells and non-transfected cells. Then, transfection efficiency was assayed by fluorescence-activated cell sorting (FACS; BD, USA).

### ELISA

Using a transwell chamber coculture experiment system, the coculture experiment was performed in the chambers of 24-well plates (Corning, USA). DMSCs (10 × 10^4^) and BMSCs were inoculated in the upper chamber, and HUVECs (1 × 10^4^) were cocultured in the lower chamber for 24 h. HUVECs without hMSCs served as a negative control. VEGF and bFGF levels in the medium of the Transwell chamber were detected via ELISA kits (Cusabio, China) according to the manufacturer's instructions (15).

### EdU Staining

Human umbilical vein endothelial cells (HUVECs) were cocultivated with DMSCs or BMSCs for 24 h using a Transwell chamber coculture experiment system. The effects of DMSCs and BMSCs on HUVEC proliferation were examined by using a commercial EdU Kit (UE, China) according to the manufacturer's protocol. Images were obtained by using a fluorescence microscope (Leika, Germany) and analyzed with ImageJ (11).

### Comparison of the Angiogenesis Abilities of DMSCs and BMSCs *in vitro*

Matrigel (100 μl/well; Corning, Standard, USA) was added to a 24-well plate (8 μm pore size; Corning, USA) and incubated for 30 min at 37°C. HUVECs (6 × 10^4^/well) were plated in Matrigel and inoculated in different media harvested from DMSC and BMSC culture media for 2 h. Images were obtained by light microscopy and analyzed by ImageJ.

### Rat Model of MI and MSC Implantation

A total of 50 adult male SD rats (200–220 g) were purchased from the animal experiment center of the Second Affiliated Hospital of Harbin Medical University and were divided into five groups: control, BMSCs, DMSCs,DMSCs + ODCshRNA and BMSCs + ODC. All procedures for animal experiments were performed in accordance with the guidelines reported in “Experimental Animal Care and Guide.” As is described in our recent study, rats were intraperitoneally injected with cyclosporin A (5 mg/kg; Novartis, Basel, Switzerland) per day from 3 days before surgery to the end of the experiment. Briefly, the rat was placed in a supine position to carry out the tracheal intubation (Artery puncture needle, 16G), the small animal inhalation anesthesia machine (Harvard Apparatus, Holliston, MA, USA) was used for anesthesia and maintaining breath, and inhaled gas was isoflurane. Through the left lateral thoracic incision, the left anterior descending coronary artery was ligated 1.5–2 mm below the left atrial appendage with a 5-0 Prolene needle. Myocardial infarction was confirmed by the whitening of the left ventricular anterior wall and apex. Ten min after ligation of the left anterior descending artery, 2.0 × 10^6^ cells from different groups were injected into the center and boundary of the infarct area.

### Cardiac Function Assessment

Cardiac function was assessed based on the left ventricular ejection fraction (LVEF) and left ventricular shortening fraction (LVFS) via echocardiography. M-mode ultrasound images were collected in the section of the left ventricular papillary muscle via an S12–4 probe (EPIQ 5; Philips). All the data were collected based on at least four consecutive cardiac cycles.

### Infarct Size Measurement

The infarct size of MI rats was measured as is previously reported (10). The ratio of the scar length and the circumference were measured by Masson's trichrome staining.

### Immunofluorescence Staining

One or 4 weeks after the operation, an immunofluorescence staining assay was performed to evaluate angiogenesis by detecting α-smooth muscle actin (α-SMA) (AF1032, Affinity) and vWF (ab6994, Abcam) expression. Moreover, transplanted cell survival was also detected by anti-cardiac troponin T (ab45932, Abcam) and anti-mitochondria (ab92824, Abcam) antibodies, according to the previous reports (15). Images were collected under an inverted fluorescence microscope (Leica, Germany).

### Statistical Analysis

Data were analyzed by using GraphPad Prism 5.0 and are presented as the mean ± SD (***n*** **=** **5**). The two groups were analyzed by *t*-test, and the multigroup comparison was analyzed with one-way ANOVA followed by Tukey's multiple comparisons *post-hoc* test. *P* < 0.05 was considered statistically significant. Each test was repeated at least thrice.

## Results

### DMSCs Promote HUVEC Proliferation and Vascularization *in vitro*

**Figure 1:**
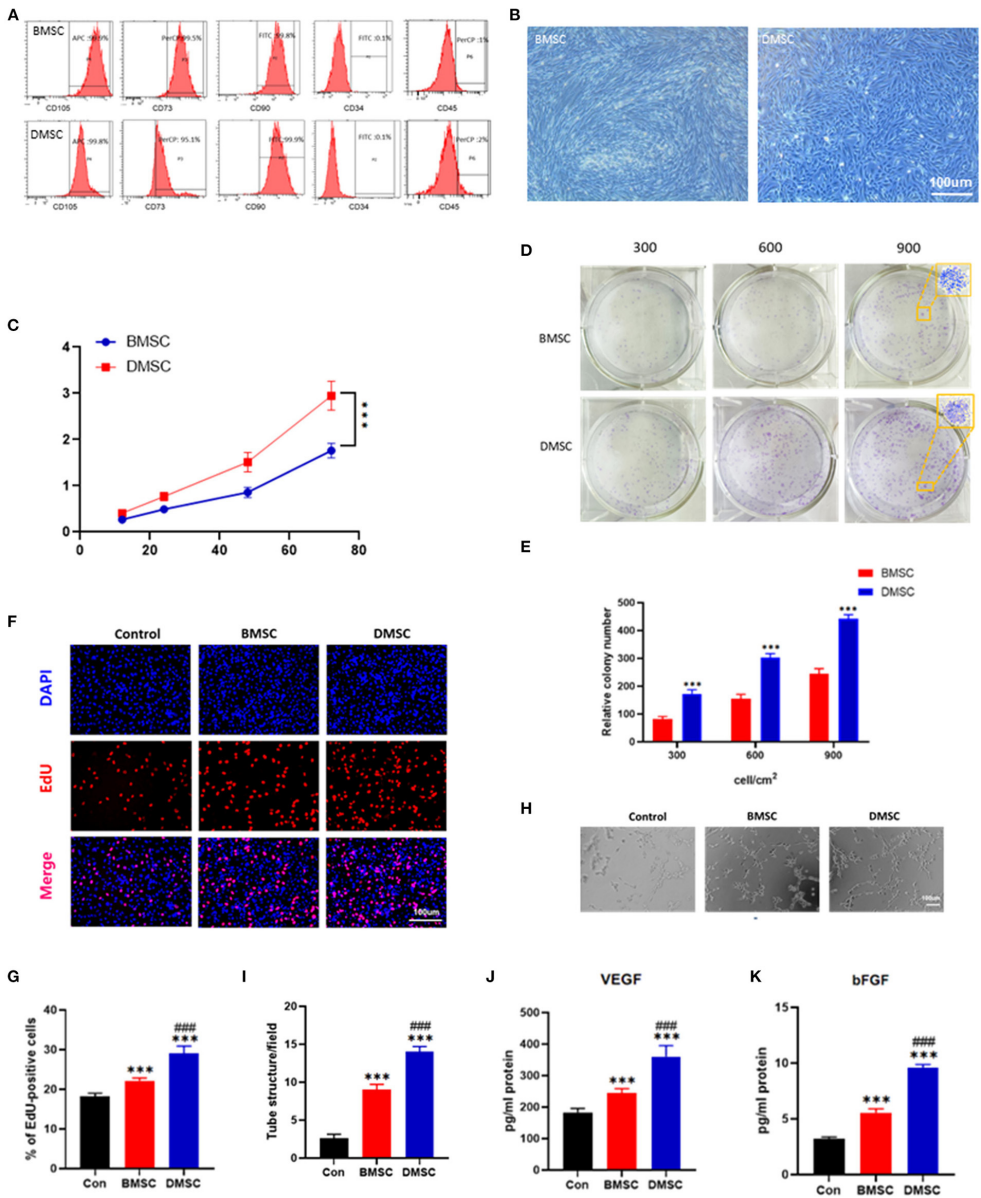
Identification of DMSCs and BMSCs and comparison of their ability to promote proliferation and vascularization *in vitro*. **(A)** Flow cytometric analysis of cell surface markers on BMSCs and DMSCs. **(B)** Morphological observation of BMSCs and DMSCs. **(C)** Cell viability of BMSCs and DMSCs. **(D,E)** Morphological and quantitative analysis of representative colonies derived from BMSCs and DMSCs. **(F)** The proliferation ability of HUVECs in response to different treatments *in vitro* as assessed by EdU staining. **(G)** Quantitative analysis of EdU-positive cells. **(H)** Representative images of tube formation of HUVECs with different treatments *in vitro*. **(I)** Quantitative analysis of tube formation. **(J,K)** Detection of VEGF and bFGF secretion levels from HUVECs under different treatment conditions *in vitro*. *P < 0.05, **P < 0.01, ***P < 0.001. ^#^*P* < 0.05, ^*##*^*P* < 0.01, ^*###*^*P* < 0.001.

Both MSCs expressed characteristic surface markers CD73, CD90 and CD105 but not CD34 and CD45 (Figure 1A). Morphologically, DMSCs exhibited fibroblast-like wall growth and a large shuttle splicing hammer shape, which is similar to the morphology of BMSCs (Figure 1B). According to the results of CCK-8 and colony experiments, the proliferation rate of DMSCs was approximately 2 times higher than that of BMSCs (P_72h_ < 0.001 for CCK-8, P_900_ < 0.001 for colony experiments; Figures 1C–E). The above results suggested that DMSCs exhibited better proliferation capability than BMSCs.

In addition, compared with BMSCs, DMSCs significantly increased HUVEC (Control) proliferation *in vitro* (P_Control−BMSC_ < 0.001, P_Control−DMSC_ < 0.001; Figures 1F,G). DMSCs effectively promoted the vascularization performance of HUVECs compared with BMSCs (P_BMSC−DMSC_ < 0.001; Figures 1H,I). Moreover, DMSCs stimulated HUVECs to produce more VEGF and bFGF, as is shown by ELISA (P_VEGF_ < 0.001, P_bFGF_ < 0.001; Figures 1J,K). These results indicated that DMSCs exhibit a more favorable capacity to boost the proliferation and angiogenesis of HUVECs than that of BMSCs.

### Ornithine and Spermine Are the Major Metabolite That Differs Between DMSCs and BMSCs

**Figure 2:**
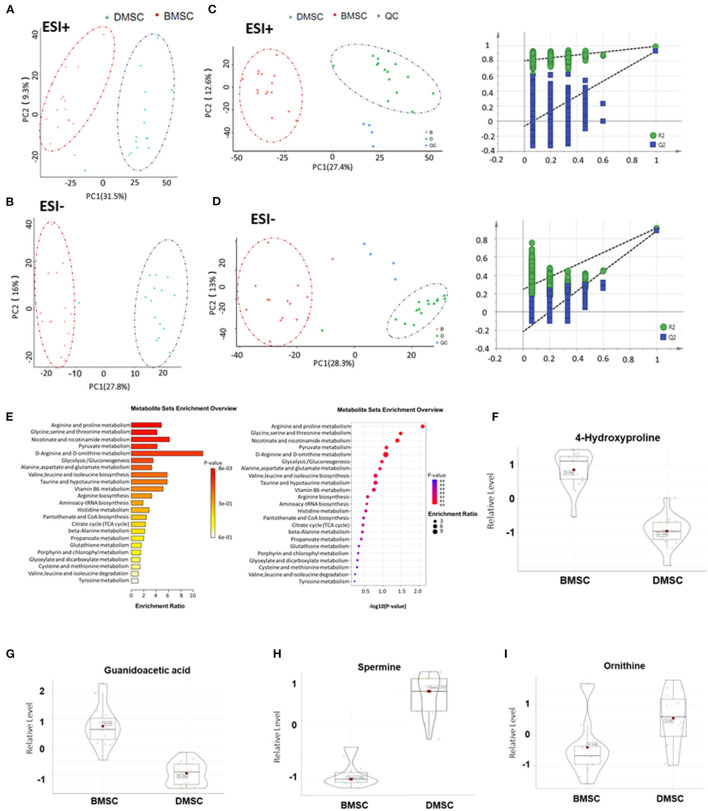
Metabolic profiles of BMSCs and DMSCs. **(A,B)** Principal component analysis score plots for discriminating BMSCs and DMSCs in ESI + and ESI- modes. **(C,D)** PLS-DA plots and validation plots for discriminating BMSCs and DMSCs in ESI + and ESI- modes. **(E)** Column chart and bubble chart of KEGG enrichment analysis of all differential metabolites. **(F–I)** Metabolite profiles of different biomarkers between epithelial BMSCs and DMSCs. Each *P*-value was < 0.01.

Unsupervised clustering showed a significant difference in metabolic features between DMSCs and BMSCs (Figures 2A,B). In addition, partial least squares-discriminant analysis (PLS-DA) showed significant differences in metabolic profiles between BMSCs and DMSCs. The displacement test showed that the Q2 cum values were lower than the original values in almost all cases (Figures 2C,D), indicating favorable stability and effectiveness of the discriminated model. According to the KEGG enrichment results for all differential metabolites, arginine and proline metabolism were the most important metabolic pathways (Figure 2E). Compared with the supernatant of BMSCs, 4-hydroxyproline and niacinamide were decreased in DMSC supernatant, whereas only ornithine and spermine were increased significantly in DMSC supernatant (Figures 2F–I). The above results suggest that the increased ornithine and spermine levels in DMSC supernatant might represent the major factor of angiogenesis promoted by DMSCs more effectively compared with BMSCs.

### ODC Inhibition in DMSCs Weakens Their Ability to Promote Angiogenesis

**Figure 3:**
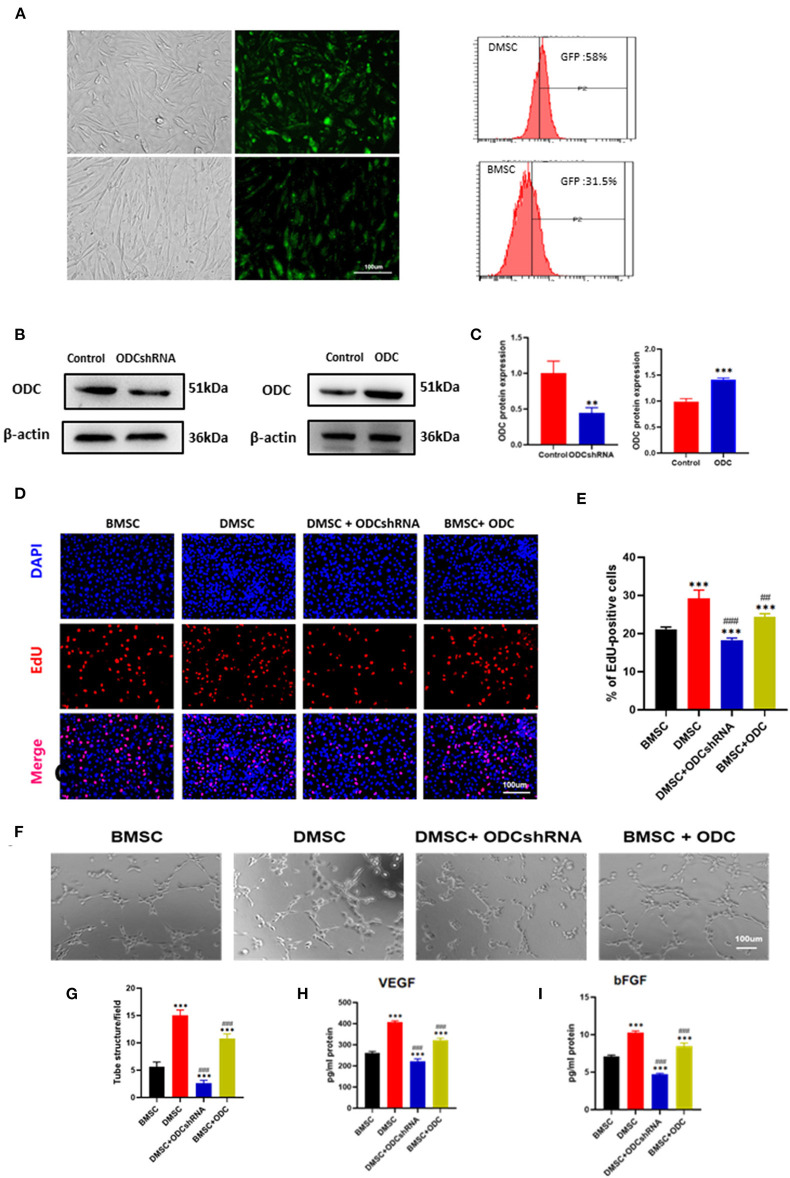
ODC inhibition partly abolished the effects of DMSCs on angiogenesis *in vitro*. **(A)** Transfection of plasmid containing GFP gene in stem cells GFP Fluorescence in DMSCs (upper row) and in BMSCs (lower row), transfection efficiency of hMSCs was detected by fluorescence-activated cell sorting. **(B,C)** ODC protein expression in DMSCs after ODC inhibition and BMSCs after ODC overexpression. **(D,E)** Representative images of DAPI/EdU staining in DMSCs or BMSCs treated with ODC shRNA or overexpression plasma for 72 h, respectively. **(F)** Representative images of tube formation in HUVECs under different treatment conditions *in vitro*. **(G)** Quantitative analysis of tube formation. **(H,I)** Detection of VEGF and bFGF secretion levels from HUVECs after different treatments *in vitro*. *P < 0.05, **P < 0.01, ***P < 0.001, Scale bar, 100 μm. ^#^*P* < 0.05, ^*##*^*P* < 0.01, ^*###*^*P* < 0.001.

The transfection of GFP plasmid could be observed under fluorescence inverted microscope, FC data showed that transfection efficiency was 58% in DMSCs and 31.5% in BMSCs (Figure 3A). After transfection of the ODC shRNA plasmid in DMSCs or ODC plasmid in BMSCs for 72 h, the protein levels of ODC were significantly down- and up-regulated in DMSCs and BMSCs, respectively (P_ODCshRNA_ < 0.01, P _ODC_ < 0.001; Figures 3B,C). As is expected, ODC inhibition in DMSCs obviously limited the proliferation of HUVEC, while ODC up-regulation in BMSCs promoted the proliferation of endothelial cells under co-cultivation conditions (P_ODCshRNA−DMSC_ < 0.01, P_ODC−BMSC_ < 0.001; Figures 3D,E). Further, whether ODC is involved in the regulation of endothelial function or not was assessed. Compared with the DMSC group, ODC inhibition for DMSCs resulted in the decrease capability of endothelial tubule formation (P_ODCshRNA−DMSC_ < 0.001; Figures 3F,G), and VEGF and bFGF secretion in HUVEC(P_VEGF_ < 0.001, P_bFGF_ < 0.001; Figures 3H,I). Conversely, ODC overexpression in BMSCs significantly promoted endothelial tubule formation (P_BMSC−ODC_ < 0.001; Figures 3F,G), and the secretion of VEGF and bFGF (P_VEGF_ < 0.001, P_bFGF_ < 0.001; Figures 3H,I). These results suggested that ODC is a critical target that promotes stem cell-mediated improvement of HUVECs proliferation and endothelial function *in vitro*.

**Figure 4:**
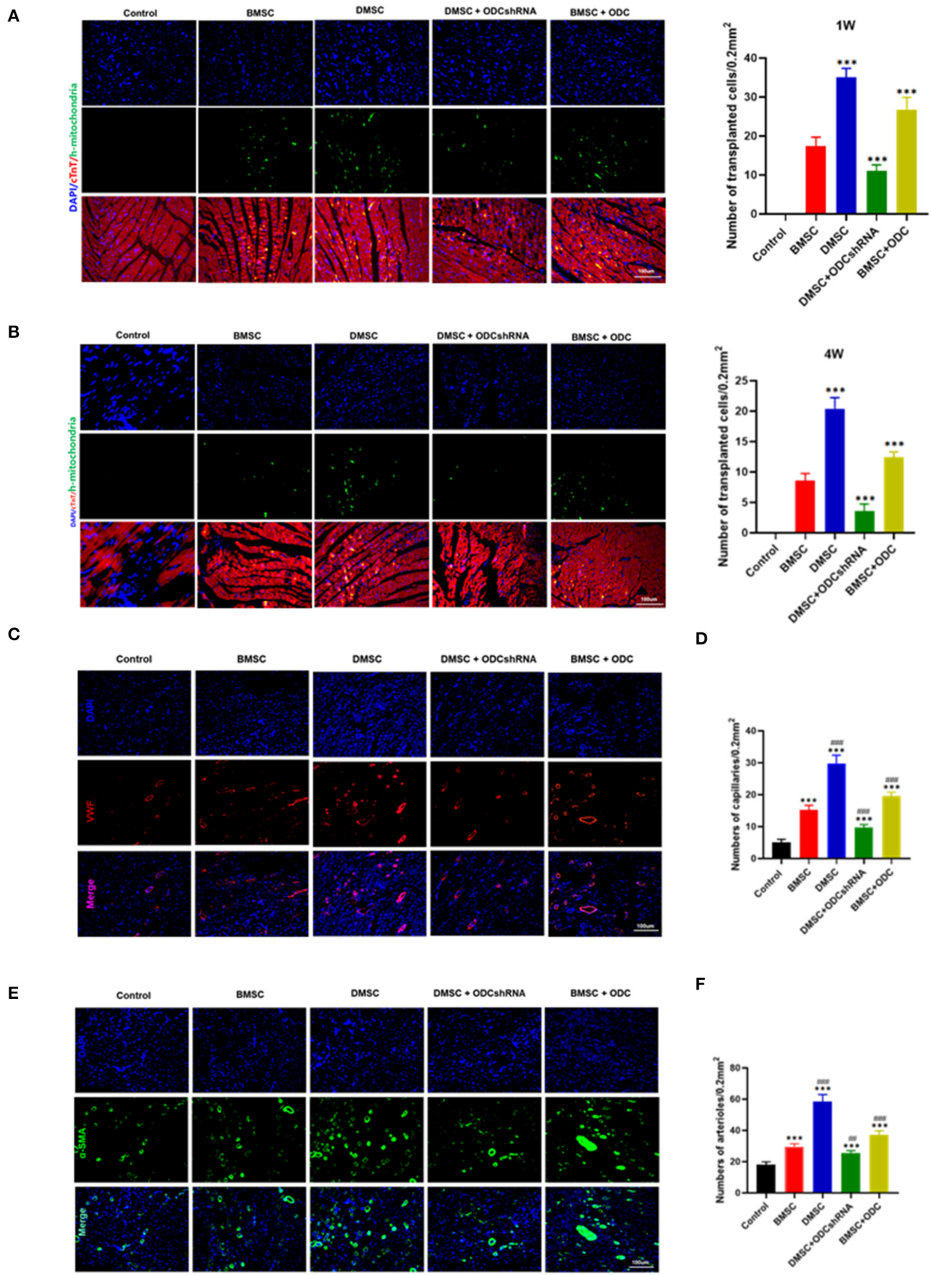
ODC inhibition partly abolished the effects of DMSCs on angiogenesis *in vivo*. **(A,B)** Anti-human mitochondrial staining showed the survival of transplanted cells for 1 and 4 weeks *in vivo*. **(C–F)** Blood vessel density determined by a-SMA and VWF staining for 4 weeks *in vivo*. ***P < 0.001, ^#^*P* < 0.05, ^*##*^*P* < 0.01, ^*###*^*P* < 0.001.

In view of the survival rate after cell transplantation, anti-mitochondrial staining results suggested that DMSCs exhibited better survival rate than that of BMSCs (P_BMSC−DMSC_ < 0.001), whereas ODC inhibition significantly reduced the survival rate of DMSCs (P_ODCshRNA−DMSC_ < 0.001), and ODC overexpression improved the BMSCs survival post transplantation in rats heart with AMI (P_BMSC−ODC_ < 0.001) (Figures 4A,B). Microvessels and arterioles in the infarct area were visualized by immunofluorescence staining for vWF and a-SMA (Figures 4C,E). The number of microvessels and arterioles in the DMSC group was significantly more than that of BMSCs groups (P_BMSC−DMSC_ < 0.001; Figures 4D,F). ODC inhibition in DMSCs reduced the density of neovascularization compared with DMSC treatment (P_ODCshRNA−DMSC_ < 0.001), while ODC up-regulation in BMSCs significantly improved the angiogenesis capability compared with wile type BMSCs transplantation (P_BMSC−ODC_ < 0.001). The results suggested that DMSCs exhibited a greater survival rate and better angiogenesis in the infarcted area of MI partly by activating ODC pathway.

### DMSCs Transplantation Improves Cardiac Remodeling and Dysfunction via the ODC-Dependent Pathway

**Figure 5:**
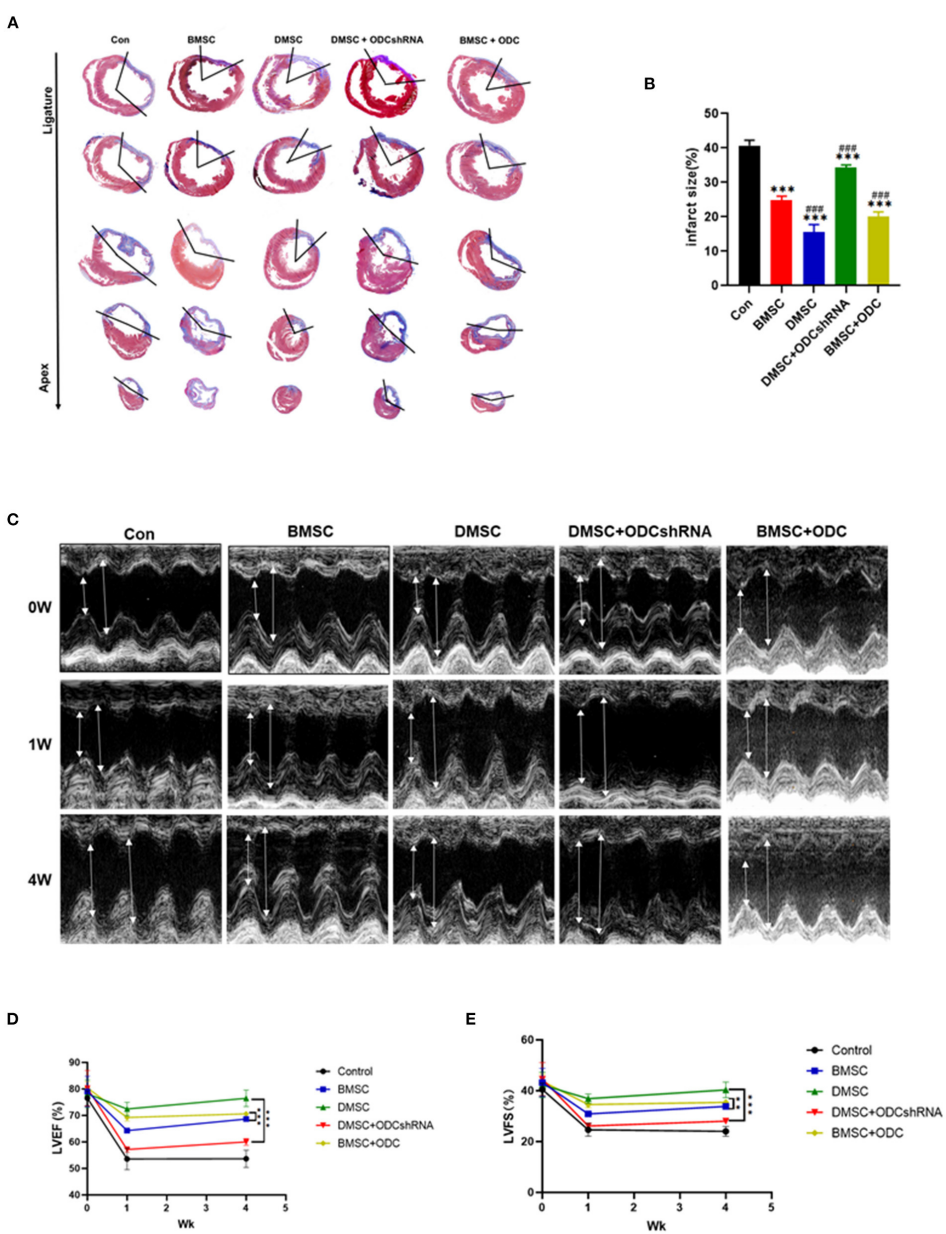
DMSC transplantation improves cardiac remodeling and dysfunction partly via the ornithine decarboxylase-dependent pathway. **(A,B)** Masson's trichrome staining to assess the infarct size 4 weeks after cell transplantation (blue = collagen; red = myocardium). Serial sections were cut at 500-μm intervals from the site of the ligature toward the apex. **(C)** Representative echocardiography images before and after MI (high lines: LVEDd; low lines: LVESd). **(D,E)** LVEF and LVFS. (ANOVA; *P < 0.01, **P < 0.01, ***P < 0.001; *n* = 5). ^#^*P* < 0.05, ^*##*^*P* < 0.01, ^*###*^*P* < 0.001.

We further explored whether ODC mediated the effect of DMSCs/BMSCs transplantation on heart function after MI. Masson staining suggested that DMSC transplantation significantly inhibited myocardial fibrosis post-MI compared with BMSCs transplantation (Figures 5A,B). However, ODC inhibition masked the benefits of DMSCs transplantation, while ODC overexpression significantly improved the therapeutic effect of BMSCs to reduce the infarcted area (P_ODCshRNA−DMSC_ < 0.001, P_BMSC−ODC_ < 0.001; Figures 5A,B). Compared with the DMSC groups, DMSCs with ODC inhibition had lower LVEF and LVFS (P_ODCshRNA−DMSC_ < 0.001, P_BMSC−ODC_ < 0.01; Figures 5D,E), as well as thinner left ventricular wall, while ODC overexpression in BMSCs significantly improved the cardiac function (Figures 5C,E). The results showed that DMSCs could delay ventricular remodeling post-MI through ODC-mediated ornithine metabolism. The long-term LVEF and LVFS after DMSC and BMSC transplantation were significantly increased compared with the control group. However, LVEF and LVFS in the BMSC + ODC group were significantly increased compared with the BMSC groups (Figures 5C–E). These results suggest that DMSC transplantation can significantly improve cardiac function post-MI partly via ODC-mediated ornithine metabolism. There was no significant between-group difference in LVESd and LVEDd (Supplementary Figure 1).

## Discussion

MI still causes more than 7 million deaths worldwide every year (16). The incidence rate of heart failure after MI was more than 50% in 5 years. Previous studies have shown that BMSCs (17), embryonic stem cells (18), iPSCs (19), CD34 + angiogenic stem cells (10) and other MSCs promote blood flow reperfusion of infarcted myocardial tissue, induce angiogenesis in infarcted myocardium, and significantly improve cardiac function. Although MSC transplantation is considered as a promising therapeutic strategy for cardiac remodeling in ischemic heart disease, the effect is not satisfactory in clinical translational research.

Once pregnant, endometrial stroma rapidly differentiates into decidua, where the vascular density increases rapidly (20, 21). Therefore, we hypothesize that MSCs from the decidua in early pregnancy may be involved in angiogenesis and vascular endothelial repairment. In addition, our previous studies initially confirmed that decidual CD34-positive stem cells significantly promote angiogenesis and improve cardiac remodeling in rats post-MI. However, the proportion of unsorted CD34-positive decidual cells was < 10%, which largely limited its clinical translation. In this study, we confirmed that CD34-negative stem cells, the main type of DMSCs, exhibit a stronger potential to promote angiogenesis and improve cardiac function in the context of MI.

Uncovering the metabolic characteristics of DMSCs and BMSCs would be helpful to understand the biological differences between both stem cells. Compared with BMSCs, arginine and proline metabolism are the most important metabolic pathways, and ornithine and spermine are crucial components of these pathways. Previous studies have confirmed that the arginine metabolism pathway is involved in angiogenesis partly by stimulating the secretion of growth hormone and insulin. Ornithine, as a basic amino acid, plays various roles in gene expression, protein synthesis, and angiogenesis of the fetus during pregnancy (22). Other reports suggest that ornithine and its corresponding metabolic pathway might play a significant role in improving placental vascular growth, wound healing and cancer treatment (23–25). Herein, our study demonstrated that ornithine metabolism in DMSCs might be the major cause of vascular endothelial proliferation and angiogenesis post-MI.

ODC catalyzes ornithine decarboxylation, which is the first rate-limiting enzyme in polyamine biosynthesis (26, 27). Reductions in ODC expression and biological activity could directly affect the production of polyamines and regulate cell proliferation and apoptosis. A previous study showed that ODC activity was a necessary condition for angiogenesis and migration of primary HUVECs (28). In this study, we further used ODC shRNA to transfect DMSCs to inhibit polyamine production, which could significantly reduce the effect of ornithine on the pro-angiogenesis of HUVECs through ODCs. We further carried out reverse verification in BMSCs and also supported this conclusion. These findings also suggest that the main mechanism by which DMSCs promote angiogenesis partly involve the regulation of ODC metabolism. Prior studies have shown that the protective effect of the ODC/polyamine system on cardiac ischemia/reperfusion injury in diabetes mellitus can be achieved by regulating the ODC/polyamine system (29–31). Our study emphasized that ODC-mediated ornithine metabolism is one of the manifest regulation pathways that distinguish DMSCs and BMSCs.

The prevalence of ischemic cardiomyopathy in premenopausal women was significantly lower than that in men, which may partly explain the advantages of DMSCs compared to BMSCs. Munira et al. showed that uterine-derived cells can home to the damaged myocardial tissue, promote myocardial repair, and improve cardiac function, indicating that uterine-derived cells might be used for the treatment of ischemic cardiomyopathy (32). Consistently, Kanwang et al. also found that EnMSCs exhibited a better cardioprotective effect than BMSCs or AdMSCs and the endometrium may be the preferred source for cardiovascular MSC therapy (5). In this study, the proliferative and clonal abilities of DMSCs were also stronger than those of BMSCs. The proliferative activity and vascular regeneration ability in the conditioned medium of the DMSC and HUVEC co-culture group were stronger than that of the BMSC and HUVEC co-culture group. Wang et al. compared the gene expression profiles, solvency, and growth factor levels of endometrial regenerative cells and bone marrow stromal cells and found obvious differences (33). Reza et al. have studied that marrow MSCs are transplanted into the myocardial infarction model of rabbits, the transplanted cells produce some angiogenesis factors and affect the internal environment, which promotes angiogenesis, reduces myocardial remodeling and improves cardiac function. Due to the uncertain results of the current clinical trials concerning BMSCs autologous transplantation in patients with cardiovascular diseases, our study further emphasizes the human DMSCs heart transplantation might be superior to BMSCs (34). We compared the differences in metabolites between DMSCs and BMSCs for the first time and found that DMSCs secreted more ornithine and spermine. DMSC transplantation improved the cardiac function and infarcted area of rats with MI. LVEF and LVFS are the most commonly used parameters to assess left ventricular systolic function (35). The insignificant difference across stem cell transplantation and control groups may be attributed to the heterogeneity in the degree of left ventricular dilatation post-MI. This individual difference could be attenuated by the ratio form, such as LVEF and LVFS (36). Histological analysis showed that DMSCs promoted the formation of microvessels and arterioles in the infarcted area through ODC metabolism.

Our study emphasizes that DMSCs is a potential choice for stem cell transplantation in the treatment of cardiac remodeling after myocardial infarction, and proves for the first time that ODC metabolism is the key target for promoting angiogenesis after myocardial infarction. Meanwhile, promoting ornithine metabolism of stem cells via targeted intervention may be a promising strategy to improve the survival and efficiency of transplanted stem cells, However, whether exists other key metabolic targets rather than ODC metabolism or not needs further research, which is also the limitation of this experiment. This study confirmed that ODC was one of the factors that the angiogenesis ability of DMSCs is better than that of BMSCs, while the mechanism is still shallow. We will further study the upstream and downstream regulation mechanism of ornithine decarboxylase spermine pathway.

These results suggest that DMSC transplantation could improve cardiac function and reduce left ventricular infarct size in the short-term and long-term recovery of MI. The improvement effect of DMSC transplantation was stronger than that of BMSC transplantation in MI, which could be attributed to the metabolites secreted by DMSCs, such as the ornithine-mediated ODC/polyamine system, which plays an important role in the treatment effects of MSCs.

## Conclusion

DMSCs transplantation exhibited a better therapeutic effect than BMSCs transplantation, which may be attributed to the more active ornithine-mediated ODC/polyamine system in DMSCs.

## Data Availability Statement

The raw data supporting the conclusions of this article will be made available by the authors, without undue reservation.

## Ethics Statement

The animal study was reviewed and approved by Ethics Committee of First/Second Affiliated Hospital of Harbin Medical University.

## Author Contributions

KC and LB performed the study and wrote the manuscript. JL and WC participated in data processing and statistical analyses. CL, EG, XQ, and XJ provided experimental technical support. HT and MH jointly conceived the study, drafted the manuscript, and all authors reviewed and approved the final version of the manuscript. All authors contributed to the article and approved the submitted version.

## Funding

This work was supported by the National Natural Science Foundation of China (No. 81770347), the National Natural Science Foundation of China (No. 81401203), the Funding for the Reserve Leader of Heilongjiang Provincial Leading Talent Echelon, the Key Laboratory of Education Ministry for Myocardial Ischemia Open Research Foundation (KF201919).

## Conflict of Interest

The authors declare that the research was conducted in the absence of any commercial or financial relationships that could be construed as a potential conflict of interest.

## Publisher's Note

All claims expressed in this article are solely those of the authors and do not necessarily represent those of their affiliated organizations, or those of the publisher, the editors and the reviewers. Any product that may be evaluated in this article, or claim that may be made by its manufacturer, is not guaranteed or endorsed by the publisher.
