# Supplementary figures and images for: Human Decidual Mesenchymal Stem Cells Obtained From Early Pregnancy Improve Cardiac Revascularization Postinfarction by Activating Ornithine Metabolism
Source: Front Cardiovasc Med. 2022 Feb 11;9:837780. doi: 10.3389/fcvm.2022.837780 (PMC8887417; doi:10.3389/fcvm.2022.837780)

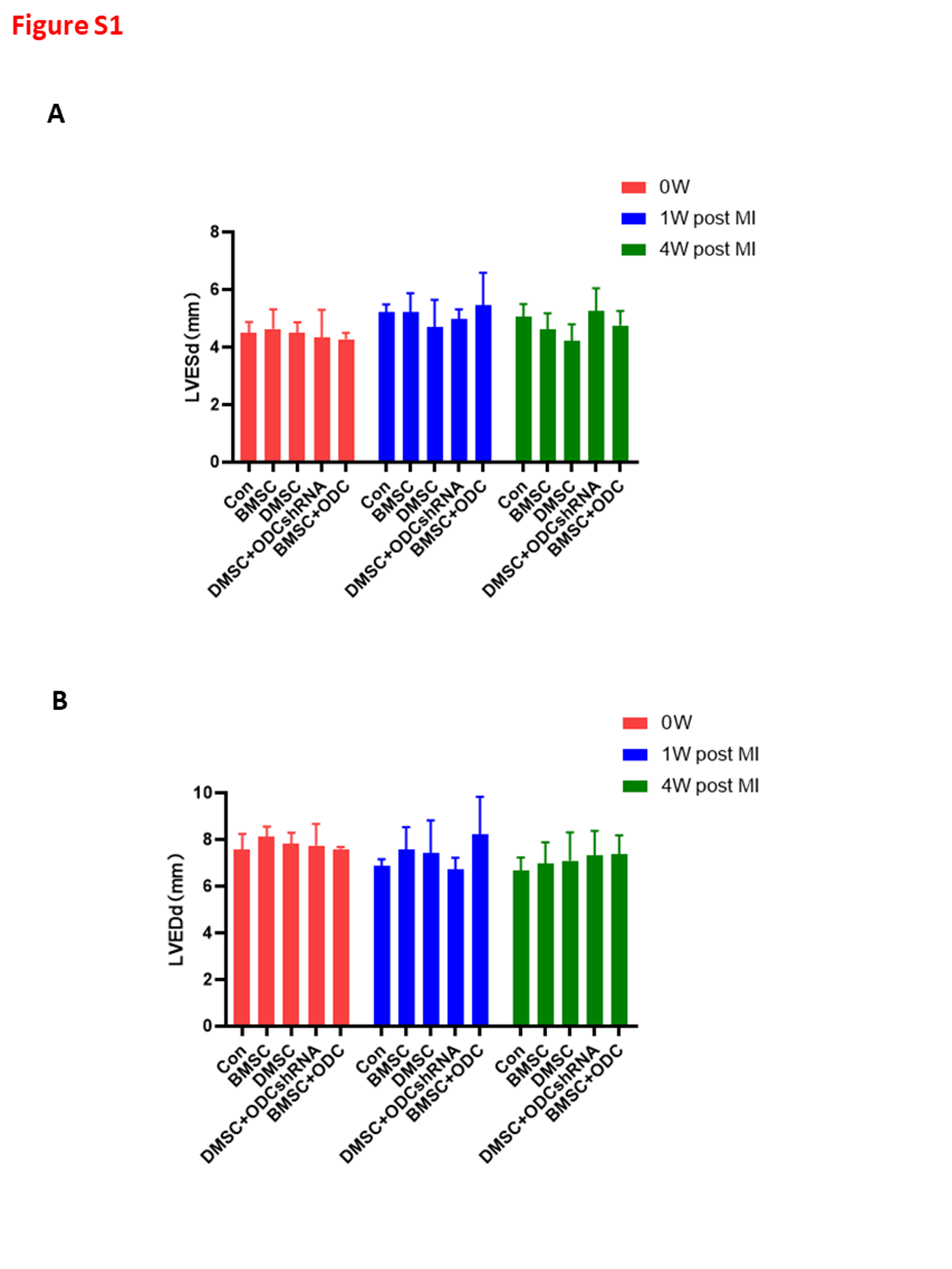

Supplement: Supplementary file 1 [file Image_1.tif]
